# Vaccination with a novel quadrivalent fusion protein protects chickens against necrotic enteritis lesions caused by *Clostridium perfringens*

**DOI:** 10.1016/j.psj.2025.105936

**Published:** 2025-10-03

**Authors:** Megha M. Manohar, Bronwyn E. Campbell, Anthony L. Keyburn, Anna K. Walduck, Robert J. Moore

**Affiliations:** a School of Science, RMIT University, Melbourne, VIC, Australia; b Australian Centre for Disease Preparedness, CSIRO, Geelong, VIC, Australia

**Keywords:** Necrotic enteritis, Vaccine, Broiler, *Clostridium perfringens*, Protection

## Abstract

Necrotic enteritis (NE), caused by *Clostridium perfringens*, is a debilitating disease that results in significant production losses in the poultry industry. Traditionally, antibiotics have been used to control NE in flocks; however, due to concerns about the potential for selection of antibiotic resistance, antibiotic residues in meat, and restrictions on antibiotic use in some regions, alternative methods to control this disease are needed. In previous studies, proteins such as NetB, the key virulence factor, and alpha toxin have been used individually as subunit vaccines, but only partial protection was induced. It appears that a single subunit antigen is insufficient to produce high levels of protection. Here, an experimental vaccine, incorporating fragments from four antigens, was designed and tested. To simplify the production and delivery of multiple recombinant antigens, a novel quadrivalent (QV) fusion protein was designed. The QV-protein vaccine and each of the individual proteins were tested as vaccine candidates in a necrotic enteritis challenge model. The birds were vaccinated subcutaneously twice and then challenged with a pathogenic strain of *C. perfringens.* The development of necrotic lesions was scored, and serum IgY antibody responses were assessed. Compared with unvaccinated birds, birds vaccinated with QV-protein had significantly lower lesion scores (*p* < 0.05). Western blot and ELISA analyses demonstrated that vaccination with the QV-protein induced antibodies specific for all four target protein fragments within the QV-protein. In conclusion, the QV-protein provides the basis for ongoing NE vaccine development.

## Introduction

Pathogenic strains of *Clostridium perfringens* are the causative agents of necrotic enteritis (NE) in chickens. For several decades, antibiotics have been used to reduce the incidence and impact of NE in poultry (Lanckriet et al., 2010). However, due to regulatory and consumer pressures resulting from concerns about the use of antibiotics in animal production, alternative disease control measures are needed. Vaccines, usually in the form of simple toxoid vaccines, have been effectively used to combat clostridial diseases in other animals, but this approach has not been successful against NE in poultry (Zaragoza et al., 2019; Abdolmohammadi Khiav and Zahmatkesh, 2021).

NE has been recognized for more than 50 years, but despite its economic impact and some success reported with various experimental vaccines (Thompson et al., 2006; Keyburn et al., 2008, 2010, 2013b; 2013a; Cooper et al., 2009; Fernandes da Costa et al., 2013), there are no highly efficacious commercial vaccines available worldwide that could be used to eradicate or control this disease. A live *Salmonella*-based vaccine is available in North America, marketed by Huvepharma, but its efficacy in the field has yet to be reported, and it is not available in other regions (Wang et al., 2022). The identification of the main virulence factor, the pore-forming toxin, necrotic enteritis toxin B (NetB), opened up new opportunities for vaccine design (Keyburn et al., 2008, 2013b). Vaccines using recombinant NetB protein (NetB), whether delivered as a conventional subunit vaccine or using a live bacterial vector, have shown some efficacy against NE, conferring partial protection and reducing the severity of intestinal lesions (Keyburn et al., 2013b; Jiang et al., 2015). Despite the identification of the key virulence factor in this clostridial disease, NE has remained a major problem for the global poultry industry (Wade and Keyburn, 2015).

The pathogenesis of NE is complex and requires the presence of secondary virulence factors in addition to NetB (Moore, 2016; Emami and Dalloul, 2021). This led to the proposal that secondary virulence factors could be incorporated into vaccines to provide multiple immune targets and improve the achievable levels of protection. Thus, several experimental multiantigen vaccines have been evaluated (Hunter et al., 2019; Katalani et al., 2020; Yuan et al., 2022). Secondary virulence factors to incorporate into a multivalent vaccine were chosen based on previous evidence of importance in strain virulence and potential vaccine efficacy. Alpha-toxin was thought to be the primary virulence factor and hence likely to be an important vaccine target before the discovery of NetB (Cooper et al., 2009). Vaccine development efforts involving alpha-toxin led to several experimental vaccines with variable protective efficacy, indicating that alpha-toxin may be a useful vaccine target even though it is not a major virulence factor in NE (Keyburn et al., 2006; Thompson et al., 2006; Hoang et al., 2008; Cooper et al., 2009). There is a strong correlation between the ability of *C. perfringens* to bind to collagen and the induction of NE (Martin and Smyth, 2010; Wade et al., 2015). Adhesion helps bacteria resist displacement from their environmental niche due to physical forces and aids the growth of bacteria in host tissue. The putative collagen adhesin, CnaA, is encoded within the VR-10B locus (Lepp et al., 2013). Disruption of the *cnaA* gene by site-directed mutagenesis led to reduced virulence in NE challenge studies (Wade et al., 2016), and this gene has been used in multicomponent vaccines with some success (Lepp et al., 2019; Yuan et al., 2022), indicating that this protein may be a useful addition to a quadrivalent vaccine. Two putative zinc metalloproteases encoded by virulent strains of *C. perfringens* have been implicated in NE pathogenesis. A protein termed “hypothetical protein” and later defined as a zinc metalloprotease, was shown to be recognized by immune serum from birds challenged with a virulent strain of *C. perfringens* (Kulkarni et al., 2006). Challenge studies with metalloprotease knockout *C. perfringens* mutant strains have shown that zinc metalloproteases influence virulence (Wade et al., 2020), and they have demonstrated some protective efficacy when used in experimental vaccines (Kulkarni et al., 2007; Katalani et al., 2020; Yuan et al., 2022).

Because of the complex nature of NE pathogenesis and the role of multiple virulence factors involved in the induction of necrotic lesions, this study adopted the strategy of investigating the vaccine efficacy of a panel of individual virulence factors and a quadrivalent (QV) protein consisting of predicted antigenic segments of four *C. perfringens* virulence factor proteins. The QV-protein incorporated fragments of a genetically attenuated form of NetB, the major virulence factor, and the secondary virulence factors alpha toxin, CnaA adhesin, and zinc metalloproteases, and was used as an injected subunit vaccine in chickens to assess immunogenicity and protection in an acute NE challenge model.

## Materials and methods

### Design and modelling of epitopes and the QV-protein

*C. perfringens* virulence factor proteins were selected as antigens of interest based on a review of the literature and existing experimental evidence. To select suitable epitope regions for use as individual proteins and for incorporation into a QV-protein, the protein sequences of the CnaA adhesin, alpha-toxin, and zinc metalloproteases were submitted to the Immune Epitope Database (IEDB) (“IEDB.org,”) for analysis. For all the predicted epitopes, bioinformatic tools were used to evaluate hydrophobicity (ExPASy ProtScale) (“ExPASy - ProtScale,”). The proteins were checked for signal peptides and transmembrane topology using Phobius (“Phobius,”). The signal peptides and transmembrane domains were removed, and regions predicted to encode epitopes were chosen. Synthetic genes were designed to express the selected protein fragments fused to a poly-histidine tag (6-His) at the C-terminal end and were synthesized by a commercial provider (GenScript).

The QV-protein was designed by linking the predicted antigenic domains of target proteins together via a peptide linker (Supplementary data 1S). The selection of the linker sequence was important for the construction of a functional chimeric protein, as the mobility and hydrophilicity of the linkers are critical considerations for ensuring the maintenance of the structural integrity of each of the selected domains (Arai et al., 2004). Spatial separation of the domains of the chimeric protein is important for the domains to fold properly and display structures similar to those found in native proteins (Arai et al., 2004). The rigid linker A(EAAAK)_4_A was used in the QV-protein to spatially separate the four domains incorporated into the design (Arai et al., 2004; Chen et al., 2013; Klein et al., 2014). Domain order was evaluated *in silico* and selected based on predicted epitope accessibility. To visualize and predict the folding of the designed recombinant proteins, Google DeepMind’s AlphaFold, an accurate artificial intelligence-based protein folding software package, was used (Jumper et al., 2021). A multimeric approach using MultiMericseqs2 of AlphaFold was used to model the three secondary virulence factors and the key virulence factor NetB, joined by the rigid helical linker (Jumper et al., 2021). After AlphaFold modelling of the QV-protein and the individual proteins, ChimeraX, with interactive tools for sequence and structure alignment, was used to visualize the predicted structures (Meng et al., 2006).

### Subunit vaccine preparation

The QV-protein and the four single antigen proteins (NetB, alpha-toxin, adhesin, and zinc metalloprotease B) were produced as polyhis-tagged recombinant proteins. Synthetic genes encoding each antigen were cloned pET-series vectors, expressed in *Escherichia coli* BL21 (DE3), and purified by immobilized nickel chromatography following the manufacturer’s instructions (Merck, document TB055). Purified recombinant proteins were formulated with a modification of the CSIRO triple adjuvant (comprising 60 % (v/v) Montanide ISA 78 VG (Seppic), 40 % (v/v) antigen combined with Quil A (InvivoGen), 3 mg/mL, and DEAE-dextran (Merck), 30 mg/mL in PBS) (Keyburn et al., 2013b). The recombinant protein adjuvant emulsion was stored overnight at 4°C before administration to the birds.

### *Preparation of C. perfringens* WER-NE36 *for in-feed challenge*

The challenge strain of *C. perfringens* used in this study, WER-NE36, a *netB^+^ tpeL^,^* strain, was prepared and used for the in-feed challenge in a similar way to that previously reported. WER-NE36, an Australian isolate of *C. perfringens,* was chosen due to its highly pathogenic nature, verified in previous animal trials (Keyburn et al., 2013b). In brief, 1-2 colonies were inoculated into 10 mL of cooked meat medium (CMM, Difco) from a culture grown under anaerobic conditions on tryptose sulfite cycloserine (TSC, Oxoid) agar and grown anaerobically for 18 h. One milliliter of this mixture was inoculated into 20 mL of fluid thioglycolate medium (FTG, Oxoid), and after growing for 18 h, 1 mL was used to inoculate 20 mL of CMM. The CMM culture was used as the final inoculum for 800 mL of FTG medium, with fresh cultures produced for use each morning and evening inoculation of feed. High-protein feed containing 50 % fishmeal and the inoculated FTG culture were mixed at a ratio of 3:4 (weight of feed/volume of culture) and fed to the birds according to the experimental design detailed below.

### Vaccination trial design and immunization schedule

**Fig. 1:**
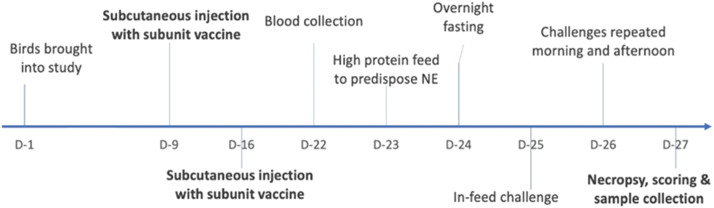
**Schedule for vaccination of chickens and in-feed NE disease induction**. The birds were immunized subcutaneously with the subunit vaccine on days 9 and 16. Blood samples were collected prior to the challenge on day 22. Lesion scoring and blood and tissue collection were performed on day 27. The numbers below the line indicate the days post hatching for each part of the study.

Eight groups of twelve commercial broilers (Ross 308, Inghams Enterprises Pty Ltd., Hatchery) were housed in a specific pathogen-free facility (CSIRO, Werribee Animal Facility) in floor pens with bedding of fresh wood shavings. The animal experiment was assessed, approved, and monitored by the Australian Centre for Disease Preparedness (ACDP) Animal Ethics Committee (approval number AEC 2026). The experimental protocol is summarized in Fig. 1. Briefly, birds were brought into the study and vaccinated twice with subunit vaccine on days 9 and 16, followed by *C. perfringens* challenge on days 25, 26 and necropsy on day 27. The birds were fed *ad libitum* an antibiotic-free and coccidiostat-free starter diet until day 22. On day 23, the feed was changed to a high-protein feed containing 50 % fishmeal to predispose the birds to NE development following the *C. perfringens* WER-NE36 challenge, as previously described (Keyburn et al., 2013b). The subunit vaccines were administered subcutaneously, with each bird given 500 µL of subunit vaccine (50 µg of individual protein vaccines and 50 µg of QV protein) at each vaccination, on days 9 and 16. The prime-boost interval of seven days, at days 9 and 16, was used to allow the development of a primary and then boosting immune response before the necrotic enteritis challenge at days 25 and 26, an age when birds are most susceptible to disease development. The immunization schedule is a compromise that is necessary when studying diseases of short-lived broilers. An adjuvant-only group was inoculated on the same schedule. No vaccination and adjuvant only control groups were challenged, and a third untreated control group remained unchallenged to demonstrate that there was no underlying necrotic enteritis in the pre-challenged birds. Animals were fasted overnight before the challenge on day 24. For the in-feed challenge, high-protein feed containing *C. perfringens* was provided for all the challenged groups in the morning and afternoon on days 25 and 26. The feed trays were cleaned, and the remaining feed was discarded prior to each subsequent feeding. The unchallenged negative control group was fed a mixture of sterile FTG media and high-protein feed.

Blood samples from the wing (brachial vein) were taken before the challenge on day 22. On day 27, all the groups were euthanized with carbon dioxide and necropsied to assess gut lesions.

### Lesion Scoring

The small intestine (duodenum to ileum) of each bird was opened and scored for macroscopic necrotic lesions. Lesions in the small intestine of necropsied birds were scored as previously reported (Keyburn et al., 2013b): 0, no gross lesions; 1, thin or friable walls; 2, focal necrosis or ulceration (1 to 5 foci); 3, focal necrosis or ulceration (6 to 15 foci); 4, focal necrosis or ulceration (16 or more foci); 5, patches of necrosis 2 to 3 cm long; and 6, diffuse necrosis typical of field cases (Keyburn et al., 2013b).

### Western Blotting and Immunoassay Detection of Antibodies

Post-challenge blood samples (day 27) were collected from all groups, and the serum was separated by centrifugation at 1000 × *g* for 10 min. To test for specificity, the recombinant proteins (300 ng) used for subcutaneous vaccination were loaded onto 4 to 20 % gradient protein gels (Mini-PROTEAN® TGX™ Precast gels, Bio-Rad) and electrophoresed at 100 V for 90 min. After SDS‒PAGE, the proteins were transferred to a PVDF membrane using an iBlot and iBlot Transfer Stack (Thermo Fisher Scientific) according to the manufacturer’s instructions. The transferred proteins were probed with pooled sera from each group, and bound antibodies were detected using goat anti-chicken IgY (*H* + *L*) horseradish peroxidase (HRP) antibody (Cat. No. A16054, Invitrogen) followed by development with Novex HRP chromogenic substrate (Cat. No. WP20004, Thermo Fisher Scientific). A polyHis-tagged protein, a fragment of a *Campylobacter hepaticus* filamentous hemagglutinin adhesin (Muralidharan et al., 2022) unrelated to clostridial proteins, was used as a test protein to assess antibody binding to the polyHis-tag.

To evaluate the IgY response in the different groups, pooled sera were analyzed via ELISA using the QV-protein and individual antigens as coating proteins. Optimization of the ELISAs was carried out at different dilutions of vaccinated serum. Preliminary titration curves indicated that a dilution of 1:2000 gave strong signals and a dynamic range that provided differentiation of antibody levels across the experimental groups; hence, 1:2000 dilution of serum was chosen to quantitatively assess all serum antibody responses. Nunc MaxiSorp™ high protein-binding capacity 96-well ELISA plates (Thermo Fisher Scientific) were used for the assay. Briefly, 0.5 µg of recombinant protein (NetB, adhesin, alpha toxin, Zmp, or QV) was used as the coating antigen (dissolved in 50 µL of 0.05 M sodium carbonate buffer, pH 9.6) and incubated overnight at 4°C. The wells were washed with PBS supplemented with 0.05 % tween 20 (PBST). The plates were then blocked using blocking solution (5 % skim milk powder in PBST) (Thermo Fisher Scientific) and incubated for 1 h at room temperature. Primary antibody solutions (1:2000 dilution of NetB, adhesin, alpha-toxin, Zmp, QV, or adjuvant group chicken sera in blocking solution) were added to each well and incubated for 1 h at room temperature. The wells were washed four times using PBST and probed with the secondary antibody goat anti-chicken Ig-Y-HRP (Thermo Fisher Scientific) diluted in blocking solution for 1 h, followed by four washes with PBS. Finally, 50 µL of 1-Step Ultra TMB-ELISA substrate (Thermo Fisher Scientific) was added to each well. The absorbance was measured after 20 min at 652 nm using a microplate reader (POLARstar Omega Plate Reader Spectrophotometer, BMG LABTECH). Every assay included triplicate samples with controls in each plate, including pooled quadrivalent serum, no challenge control pooled serum, adjuvant control pooled serum, and blanks. The control immunoassay results were recorded for every assay to check for consistency, and the blank values were used in the calculations. The individual bird serum samples from the nonchallenge group and adjuvant group were checked for specificity for QV and individual proteins. If any sample was found to have differing values among the triplicates, the assay was repeated to confirm the true value and then incorporated into the statistical analysis.

### Statistical Analysis

Statistical analysis was performed using GraphPad Prism (v9.3.1) with Kruskal‒Wallis one-way ANOVA with Dunn’s multiple comparisons post hoc test for lesion scoring and ELISA results and the Mann‒Whitney U test for FACS data. A value of *p* < 0.05 was considered to indicate statistical significance.

## RESULTS

### Epitope Prediction of Individual and QV Vaccine Candidates

To increase the chances of obtaining satisfactory expression levels of each of the individual proteins, the sequences were examined for predicted signal sequences and transmembrane domains so that they could be excluded from the expressed protein expression constructs. For the expression of CnaA as an individual antigen, the region from amino acids 30 to 688 was used. For alpha toxin, amino acids 29-398 of the native protein were used as the individual protein antigen. For NetB, amino acids 30 to 292 of the native protein were used as the individual protein antigen. Native ZmpB is a very large protein (1687 aa), and thus, for this protein, less than a third was used (aa 1080 to 1528) for the individual protein antigen.

For the quadrivalent vaccine candidate, small fragments of the four individual proteins were selected based on epitope mapping results obtained using the IEDB and from previous protection results from NE challenge studies (Kulkarni et al., 2006, 2007; Zekarias et al., 2008; Lepp et al., 2010; Keyburn et al., 2013b; Noach et al., 2017; Katalani et al., 2020). In all cases, the smaller fragment of each protein used to design the QV-protein was fully contained within the larger fragments used for the expression of individual proteins. The protein sequence of the collagen adhesin (CnaA) (GenBank KT749987.1) was submitted to the IEDB, and predicted epitopes were identified. A transmembrane domain in the region 669-690 aa of CnaA was detected by Phobius, and this region was excluded from consideration for vaccine design. The amino acid sequence from coordinates 440 to 640 of the native protein was selected for inclusion as the CnaA fragment in the QV-protein. In the study by Zekarias et al. (2008), the carboxy terminal domain of alpha toxin from *C. perfringens* was used as a vaccine target (Zekarias et al., 2008). The protein sequence of alpha toxin (PlcC) (GenBank M24904.1) was submitted to the IEDB for *in silico* epitope prediction; the potential immunogenicity of a fragment used in a previous study was confirmed (Zekarias et al., 2008). The C-terminal domain of the alpha toxin, from amino acid sequence 248 to 370 of the mature protein, was chosen as the fragment for inclusion in the QV-protein. The protein sequence for zinc metalloprotease (ZmpB) (GenBank new locus tag CPF_RS07240, old locus tag CPF_1489) was from the *C. perfringens* strain ATCC 13124 genomic sequence (Noach et al., 2017). The protein sequence for ZmpA was obtained from Lepp et al. (2010) (GenBank locus tag CP4_3468) from the *C. perfringens* strain CP4 plasmid pCP4netB pathogenicity locus 1 genomic sequence (Lepp et al., 2010). Kulkarni et al. (2006) discovered a hypothetical protein (HP) in *C. perfringens* strain 13 (GenBank accession no. 18144943), which was subsequently designated Zmp1 (Kulkarni et al., 2006). Pairwise alignment via BLASTP (“BLAST: Basic Local Alignment Search Tool,”) revealed that ZmpA and ZmpB have 64 % identity (Wade et al., 2020), and HP/Zmp1 from Kulkarni *et al*. (2006) shares 99 % identity with ZmpB and 65 % identity with ZmpA. These zinc metalloproteases contain a gluzincin-like motif that is responsible for zinc binding and acts as the catalytic core; this key region was included in the final design. The amino acid sequence 1170 to 1355 of ZmpB, which shares a high level of homology with the equivalent region in ZmpA, was chosen as the fragment for inclusion in the QV-protein. The amino acid sequence 2 to 192 of the attenuated mature NetB sequence (Keyburn et al., 2013b) was selected as the fragment for use in the QV-protein. The selected fragments of NetB_2-193_, CnaA_440-640_, alpha toxin_248-370_, and ZmpB_1170-1355_ were concatenated with an alpha-helical linker peptide, A(EAAAK)_4_A, separating each section. The designed QV-protein has a molecular weight of 77 kDa. The amino acid sequence is detailed in the supplementary data file, and the quality of the QV-protein used in the vaccinations is shown in Supplementary Fig. 1.

### Structural modelling of the QV-protein with rigid linkers

**Fig. 2:**
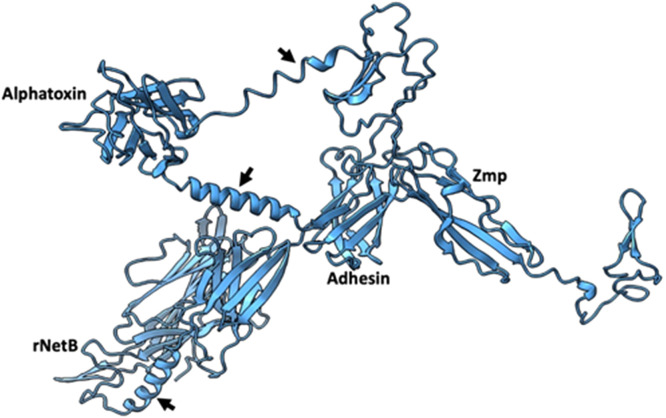
**AlphaFold multimeric modeling of the QV-protein**. The quadrivalent protein contains fragments of recombinant NetB, adhesin, alpha-toxin, and zinc metalloprotease, as labeled, and joined by the rigid alpha helical-linker A(EAAAK)_4_A, as indicated by arrows. The rigid linkers fold separately from the domains, and the model shows minimal interactions between the protein fragments.

The results of structural modelling of the QV-protein sequence using AlphaFold are shown in Fig. 2. Structural modelling revealed that rigid linkers produced spatial separation, limited interactions, and no hidden domains in the vaccine epitopes of the four constituent virulence factor fragments that were combined into the QV-protein design. Based on the structural predictions, the QV-protein was predicted to display the selected epitopes in conformations were similar to the equivalent section in the mature native proteins and that were likely to be immunogenic.

### The QV-protein-vaccinated chickens produced antibodies that bound to each of the individual component antigens

**Fig. 3:**
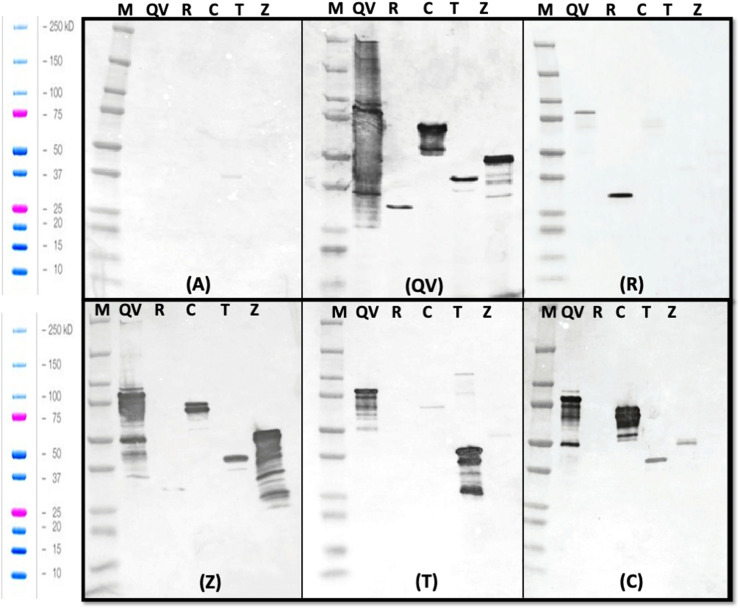
**Western blot of pooled sera against recombinant proteins**. The recombinant proteins were probed with serum of groups vaccinated with; A – adjuvant only, B – QV-protein vaccinated, C – NetB vaccinated, D – Zmp vaccinated, E – alpha-toxin vaccinated, or F – adhesin vaccinated. Lane M: Precision Plus Protein Dual Xtra (Bio-Rad); Lane QV: QV-protein (77 kDa); Lane N: NetB (26 kDa); Lane C: adhesin (65 kDa); Lane T: alpha-toxin (38 kDa); Lane Z: zinc metalloprotease (47 kDa).

Serum samples collected from all groups at necropsy (day 27) were tested by western blot analysis for specificity against the individual recombinant proteins and the QV-protein. Fig. 3 shows the specificity of the serum from each vaccinated group. Pooled sera from the adjuvant control group did not bind strongly to any of the recombinant proteins (Fig. 3A). The weak positive reaction to the alpha toxin band (lane T) may represent low-abundance cross-reactive antibodies induced by an endogenous clostridial strain that might have been present in the gut microbiota and was observed in all groups to varying degrees. NetB- and alpha toxin-vaccinated group sera bound to the QV-protein and the corresponding vaccinated protein (Fig. 3C and 3E), generating bands at the expected size and some likely degradation products. Sera from the Zmp- and adhesin-vaccinated groups recognized antigens not only of the vaccinated individual protein and QV-protein, as expected, but also other proteins, particularly alpha toxin, as discussed above, as well as faint binding to the Zmp protein (Fig. 3D and 3F). The serum samples from the QV-protein group recognized the QV-protein and all the individual proteins (Fig. 3B).

**Fig. 4:**
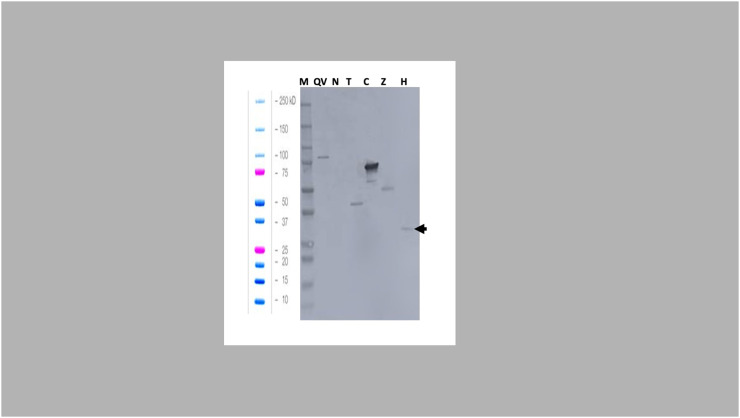
**Western blot of pooled sera of adhesin-vaccinated birds against recombinant proteins and an unrelated His-tagged protein**. Lane M: Precision Plus Protein Dual Xtra (Bio-Rad); Lane QV: QV-protein (77 kDa); Lane R: NetB (26 kDa); Lane C: adhesin (65 kDa); Lane T: alpha-toxin (38 kDa); Lane Z: zinc metalloprotease (47 kDa); Lane H: NE trial unrelated FHA His-tagged protein (35 kDa). The recombinant proteins were probed with serum from the group vaccinated with adhesin and found to bind to an unrelated his-tagged protein (black arrow) in Lane H, alpha-toxin protein in Lane T, and ZmpB protein in Lane Z.

The cross-reactivity of the Zmp- and adhesin-vaccinated group sera to other single recombinant proteins (NetB and alpha toxin) was unexpected. We hypothesized that this may have been due to the antibodies being raised to the polyHis tag that was fused to each recombinant protein. To test this hypothesis, the sera from the adhesin-vaccinated group were subjected to western blotting to test reactivity against an unrelated polyHis-tagged protein, a fragment of filamentous hemagglutinin adhesin (FHA) (Fig. 4). The results confirmed a low cross-reactivity to the polyHis tag, and the intensity of the cross-reactive band was not strong compared to that of the adhesin band. Therefore, birds vaccinated with recombinant Zmp and adhesin produced a weak immune response against the polyHis tag, resulting in the additional bands observed in Fig. 3, Fig. 4 but stronger immune responses against the *C. perfringens* proteins.

**Fig. 5:**
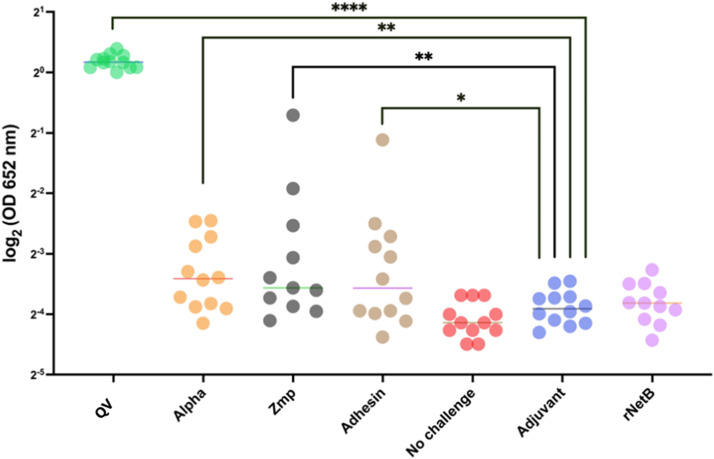
**Serum IgY responses of chickens immunized with individual subunit vaccine candidates to the QV-protein**. Individual sera from each group were tested against the QV-protein. QV-protein vaccinated birds presented significantly greater anti-QV-protein IgY levels than groups vaccinated with individual proteins. The absorbance was plotted as log_2_ values on the Y-axis with the type of subunit vaccine used for immunization on the X-axis. The horizontal lines represent the group averages of the absorbance values. Statistical significance was assessed using Kruskal‒Wallis one-way ANOVA with Dunn’s multiple comparisons post hoc test. The most important results are indicated in the figure. The groups vaccinated with recombinant protein were also significantly different from the unchallenged control group. **** p value < 0.0001, *** p value < 0.001, and ** p value < 0.01.

To further evaluate the IgY immune responses, ELISAs were performed. First, the serum of birds vaccinated with the individual antigens, the QV-protein, and the control groups was assayed against the QV-protein (Fig. 5). Statistical analysis of ELISA absorbance values indicated a significant response in the groups vaccinated with QV-protein (*p* < 0.0001), alpha-toxin and Zmp (*p* < 0.01), and adhesin (*p* < 0.05). The specific IgY responses in the birds immunized with NetB did not significantly differ from those in the adjuvant or negative control groups, indicating that the IgY produced in response to NetB vaccination did not recognize the QV-protein in the ELISA (Fig. 5) but was sufficient to provide a clear positive signal by western blotting (Fig. 3C). The results of immunoassays of serum IgY responses against the QV-protein showed that birds vaccinated with individual proteins produced an immune response that recognized the corresponding epitopes in the QV-protein, with the exception of the NetB-vaccinated birds. The QV-protein-vaccinated group had the highest serum IgY response to QV-protein (Fig. 5).

**Fig. 6:**
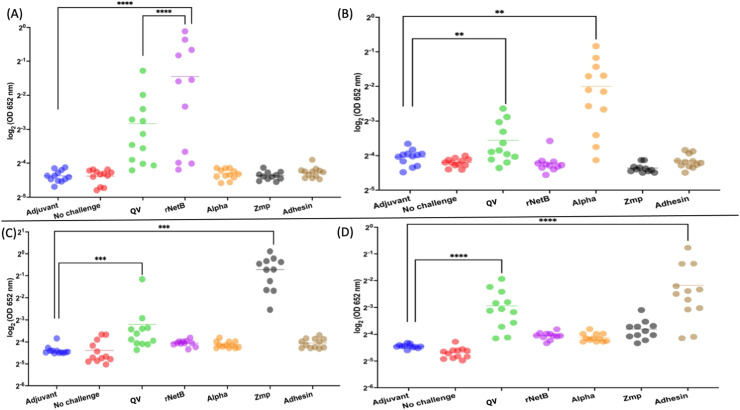
**Serum Ig-Y responses of chickens to (A) NetB, (B) alpha-toxin, (C) zinc metalloprotease and (D) adhesin**. IgY antibody responses were measured by ELISA using different individual protein coatings as indicated by (A), (B), (C) and (D) against a 1:2000 dilution of chicken serum. The absorbance was plotted as log_2_ values on the Y-axis with the type of subunit vaccine used for immunization on the X-axis. The horizontal line represents the average absorbance values. Statistical significance was assessed using Kruskal‒Wallis one-way ANOVA with Dunn’s multiple comparisons post hoc test. **** p value < 0.0001, *** p value < 0.001, and ** p value < 0.01.

Further ELISAs demonstrated that the antibodies raised by QV-protein-vaccinated birds could recognize the NetB protein (Fig. 6A). Therefore, although antibodies raised to the individual NetB protein could not efficiently recognize the QV-protein in the ELISAs, antibodies raised to the QV-protein could recognize NetB protein. The QV-protein also elicited significant serum IgY responses that could recognize each of the other individual proteins and thus demonstrated that each of the four domains in the QV-protein were sufficient to produce a quadrivalent immune response (Fig. 6 (A to D)).

### The QV-protein vaccine protected chickens from the development of NE lesions in the small intestine

**Fig. 7:**
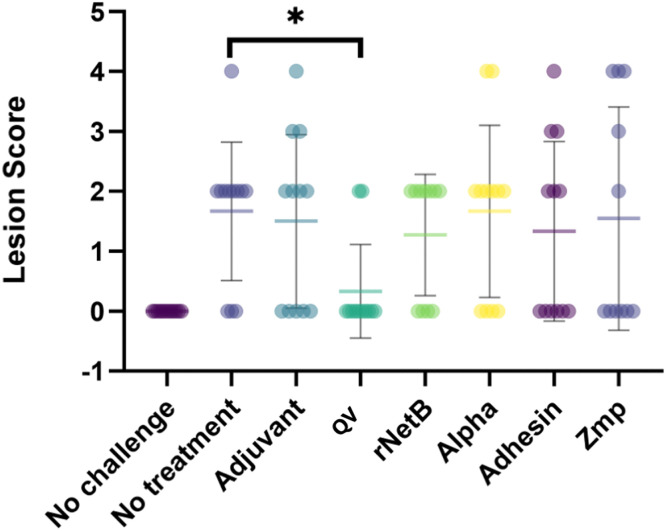
**Individual lesion scores of chickens immunized with subunit vaccines**. Mean (+-SD). Intestinal lesion scores from the small intestine (duodenum to ileum) were scored as previously reported (Keyburn et al., 2013b). The QV-vaccinated group had a significantly lower average lesion score than the untreated group (*p* < 0.05).

All chickens, except those in the no-challenge control group, were challenged with virulent *C. perfringens* to induce NE and thus determine whether the immune responses induced by the vaccine formulations could provide protection from NE lesion development. Lesion formation in the QV-protein-vaccinated group was significantly different from that in the no-treatment control group (p value <0.05) (Fig. 7), and the associated multiple comparison test showed p values of 0.027 for the QV-protein/no-treatment comparison and 0.069 for the QV-protein/adjuvant comparison. Birds vaccinated individually with NetB and the secondary virulence factors were not protected from NE lesion formation.

When comparing the severity of disease, neither the average lesion score for a group nor the number of affected birds provided a complete representation of efficacy. In an attempt to consider both lesion score data and the number of animals affected, a new hybrid measure, the Group Lesion Score (GLS), is proposed; this score is calculated by multiplying the average lesion score for a group by the number of birds in the group with lesions. This measure aims to provide a single measure for disease that captures both severity and overall occurrence. The QV-protein-vaccinated GLS was 0.66 (average lesion score × number of affected birds = 0.33 × 2), and the scores were 15.03 (average lesion score × number of affected birds = 1.67 × 9) for the untreated group and 10.5 (average lesion score × number of affected birds = 1.5 × 7) for the adjuvant-only group (Table 1). The percent reduction was calculated by comparing the vaccinated GLS with the untreated group and adjuvant only GLSs. In the QV-protein-vaccinated group, there was a 96 % reduction in GLS after challenge compared to that in the untreated group and a 94 % reduction in GLS compared to that in the adjuvant-only group. Using the traditional approach of comparing average lesion scores, the level of protection achieved in the QV-protein-vaccinated group was 80 % compared to the untreated control group or 77 % compared to the adjuvant control group. Body weights were recorded for all birds. No significant differences were noted due to the short interval between challenge and necropsy and the small number of birds used per group – significantly larger group sizes are generally required to assess weight differences between groups. Animal ethics considerations meant the use of larger groups was not warranted, as lesion scoring was sufficient to demonstrate vaccine efficacy.

**Table 1:** NE lesion scores for treatment groups in the vaccination trial.

	Treatment Group							
	QV-protein	No treatment	Adjuvant	No challenge	NetB	Alpha toxin	CnaA adhesin	Zmp
**Average lesion score**	0.33	1.27	1.50	0	1.27	1.67	1.50	1.55
**No. of birds with lesions**	2/12	9/12	7/12	0/12	7/11	8/12	6/12	5/11
**GLS**^1^	0.66	15.03	10.50	0	9.70 2	13.36	9.00	8.45 2

1 GLS, Group Lesion Score.

2 different group size adjusted values to account for the need to cull one bird each from the NetB and Zmp groups on day 9, before the first vaccination, due to failure to thrive.

## Discussion

The need for an effective vaccination strategy to ameliorate the impact of NE in broiler flocks is evidenced by the growing number of studies that have reported on this topic. It is difficult to draw definitive conclusions from these studies because there are great variations in the study designs, including different vaccination schedules, dosages, adjuvants, different NE challenge models, disease scoring systems, and different immunological parameters (Moore, 2024). The present study evaluated the protection of chickens from NE challenge using a quadrivalent fusion protein subunit vaccination approach. This approach was taken because the accumulating evidence from experimental vaccine trials indicated that single-subunit vaccines are unlikely to provide high levels of protection from disease development. The study compared subunit vaccines both as individual antigens and in the form of a quadrivalent fusion protein (QV-protein) that was composed of epitopes from multiple virulence factors. The QV-protein vaccine elicited high levels of antibodies that recognized both the QV-protein and the individual proteins and provided good protection against the development of NE lesions in the small intestine. Immunization with individual antigens did not provide any protection against NE lesion formation. These findings further support the idea that immune responses directed against multiple *C. perfringens* targets are far more likely to produce a protective response than when only single antigens are used.

Previously, a trivalent fusion protein, NAM, which used fragments of NetB, alpha-toxin, and zinc metalloprotease (NetB_146-322_, alpha-toxin_284-398_ and Zmp_698-1022_), was assessed for its ability to induce immune protection against lesions in a NE challenge trial (Katalani et al., 2020). The candidate trivalent vaccine protein was expressed in tobacco plants, was delivered via subcutaneous or oral routes and induced significant protection against an NE challenge (Katalani et al., 2020). The QV-protein used in the present study contained different fragments of NetB_2-193_, alpha-toxin_248-370_, and ZmpB_1170-1355_; and additionally, a segment from the CnaA_440-640_ adhesin, each joined by the rigid alpha-helical linker peptide A(EAAAK)_4_A, and with a polyHis-tag region to enable purification and detection. The CnaA adhesin was previously shown to be an important virulence factor (Wade et al., 2016) and has demonstrated potential as a vaccine antigen when used alone and in combination with other antigens (Lepp et al., 2019; Yuan et al., 2022), and a synthetic multiepitope protein containing predicted T- and B-cell epitopes has been designed but not yet tested in an NE challenge trial (Chundru et al., 2025). One compromise that was made in designing and producing the QV-protein was that only fragments rather than complete versions of the selected virulence proteins were used. This approach was used to ensure that the recombinant fusion protein could be efficiently produced. A fusion protein that used the complete sequence of each of the four antigens would be very large, contain secretion signal sequences and transmembrane domains, and would almost certainly be very difficult to express and produce in sufficient quantities. The choice of protein fragments used is likely to have a significant effect on the ease of recombinant production and on the protective efficacy of induced antibody responses. The fragments of the virulence proteins used in the QV-protein were selected based on bioinformatic analysis that aimed to identify likely epitopes, and the predicted structure of the QV-protein was assessed to confirm that the rigid linkers enabled each of the antigen regions to fold in a conformation that resembled the conformation predicted for each of the whole native proteins. This strategy appeared to be largely successful because antibodies raised to three of the four antigen fragments within the QV-protein recognized both the QV-protein and each of the individual recombinant proteins; conversely, antibodies raised against the individual proteins recognized the QV-protein. An exception was observed for the NetB protein. Antibodies induced in response to vaccination with NetB did not recognize the QV-protein in the ELISAs, although the QV-protein was detected by western blotting with the sera from the NetB vaccinated group. Antibodies induced by vaccination with the QV-protein did recognize the NetB protein. Further experimentation and analysis are needed to determine the basis of these phenomena, but these findings may be related to the folding of the proteins under different conditions; for example, the denatured protein displayed on SDS-PAGE western blots compared with the likely more native structure that could be present in the ELISA format.

An alternative approach to a quadrivalent vaccine would be to express each antigen separately and then combine the separately produced recombinant proteins into a single vaccine formulation. This approach has been employed in other studies (Lepp et al., 2019; Yuan et al., 2022), but it was not suitable for addressing the long-term goals of the present study. The use of conventional injectable vaccines is a valuable tool for assessing antigens that might be valuable for use in a vaccine directed against chicken NE, but this form of vaccination is not a useful option for large-scale commercial use in most broiler industries worldwide because of the handling costs involved. Alternative formulation and delivery strategies are required for low-cost mass application to broilers. Future work will develop live bacterial delivery vectors that can deliver the QV-protein. This approach to vaccine design is most feasible when a single recombinant protein is to be delivered; hence, the quadrivalent QV-protein rather than the separate expression of four individual antigens is needed. In the future, it may be possible to enhance the efficacy of live vector-mediated delivery of QV-protein by incorporating other proteins into the design that can enhance immune responses (Manohar et al., 2022).

Using the QV-protein approach and careful selection of immunogenic fragments, a 96 % reduction in GLS was achieved with the experimental vaccine compared to the unvaccinated control group, or a 94 % reduction compared to the adjuvant control group. This equates to an 80 % and 77 % reduction in lesions using the traditional average lesion score method of assessment against no treatment or adjuvant controls, respectively. The epitopes used in the QV-protein were selected from proteins that have a considerable record of use in vaccination trials and three of the four proteins (NetB, CnaA, and Zmp) have been shown to be important for *C. perfringens* pathogenesis using defined gene knockouts (Keyburn et al., 2008; Wade et al., 2016, 2020). However, a variety of other proteins, including ArcB, TmpC, EntB, PFOR, and FBA have also shown efficacy in vaccination trials and should be considered for inclusion in ongoing experiments to define the most efficacious multivalent antigen (Kulkarni et al., 2007; Li et al., 2024; Zhang et al., 2025).

Previous work has demonstrated that the vaccination of broiler breeder hens can provide some degree of protection to broiler chicks (Keyburn et al., 2013a). This finding suggested that egg derived IgY antibodies play a role in immunological protection from NE. However, in experimentally vaccinated chicks, no correlation has been reported between *C. perfringens* antigen-specific IgY antibody titers and the level of disease observed in vaccinated birds (Keyburn et al., 2013b). Therefore, the immunological mechanisms involved in delivering the protection observed with some experimental vaccines are unclear. In future work, it would be useful to follow immune responses, including IgA and cellular responses, over a more extended period, for example, throughout the whole life of broilers (approximately 6 weeks for conventional commercial chickens), although the most vulnerable period for broiler susceptibility to NE is normally the 2-to-4 week period post hatching.

These findings support the hypothesis that a multivalent approach is an effective choice for combating NE via vaccination. However, vaccine delivery by direct subcutaneous vaccination, as used in this experimental proof-of-principle study, is not feasible for the bulk of the global broiler population. Other more appropriate vaccination methods, such as *in ovo* vaccination, maternal vaccination, or delivery by live bacterial or viral vectors, need to be developed to deliver the QV-protein. Engineered live bacteria can be used as tools for the delivery of vaccine antigens to the gut mucosa (Medina and Guzmán, 2001). Direct delivery of vaccine antigens to the gut can potentially induce a strong mucosal immune response. Mucosal immune responses are likely to be the most appropriate type of immune response for protection against pathogens that invade mucosal surfaces (Goh et al., 2012). To further improve the memory immune response, delivery of the quadrivalent vaccine via a live bacterial vector has excellent potential for delivery to broiler flocks, as it can be administered in feed or water. Delivery of live vaccines via the oral route enhances the likelihood of mucosal immune responses.

In conclusion, a quadrivalent antigenic protein delivered by the subcutaneous route has been shown to induce antibody responses and protection against lesion formation in an acute NE challenge model. The quadrivalent antigenic protein described in this study provides a powerful tool for ongoing vaccine development.

## Declarations

### Ethics approval

The animal experiment was assessed, approved, and monitored by the Australian Centre for Disease Preparedness (ACDP) Animal Ethics Committee (approval number AEC 2026).

### Availability of data and materials

All the data are included in the paper.

### Competing interests

RJM and ALK are inventors on a patent on NetB and its use in vaccines: Clostridial Toxin NetB. US Patent no. 8,263,088 B2. We declare no other competing interests.

### Funding

Funding was provided by an Australian Research Council Linkage grant (LP160101044). The funder had no role in the study design or the collection and interpretation of data, or manuscript preparation and submission.

## CRediT authorship contribution statement

**Megha M. Manohar:** Conceptualization, Methodology, Resources, Investigation, Formal analysis, Writing – original draft. **Bronwyn E. Campbell:** Conceptualization, Methodology, Resources, Investigation, Formal analysis, Writing – review & editing. **Anthony L. Keyburn:** Conceptualization, Methodology, Resources, Investigation, Formal analysis. **Anna K. Walduck:** Funding acquisition, Resources, Investigation, Formal analysis, Writing – review & editing. **Robert J. Moore:** Funding acquisition, Supervision, Conceptualization, Methodology, Resources, Investigation, Formal analysis, Writing – review & editing.

## Disclosures

The authors declare the following financial interests/personal relationships which may be considered as potential competing interests: Megha Mano Manohar reports financial support was provided by Australian Research Council. Megha Mano Manohar reports a relationship with Commonwealth Scientific and Industrial Research Organisation that includes: employment. Robert J Moore has patent #8,263,088 B2 issued to US Patent. If there are other authors, they declare that they have no known competing financial interests or personal relationships that could have appeared to influence the work reported in this paper.

## Appendix Supplementary materials

Image, application 1

## Footnotes

Supplementary material associated with this article can be found, in the online version, at doi:10.1016/j.psj.2025.105936.
