# Supplementary material for: Vaccination with a novel quadrivalent fusion protein protects chickens against necrotic enteritis lesions caused by Clostridium perfringens
Source: Poult Sci. 2025 Oct 3;104(12):105936. doi: 10.1016/j.psj.2025.105936 (PMC12547217; doi:10.1016/j.psj.2025.105936)
Supplement: Supplementary file 1 [file mmc1.docx]

**Supplementary data**

**1S Quadrivalent protein**

The following sequence was submitted for synthesis,

ATG start codon**—Multimer with rigid linker**--His tag--Stop codon

M**SELNDINKIELKNLSGEIIKENGKEAIKYTSSDTASHKGWKATLSGTFIEDPHSDKKTALLNLEGFIPSDKQIFGSKYYGKMKWPETYRINVKSADVNNNIKIANSIPKNTIDKKDVSNSIGYSIGGNISVEGKTAGAGINASYNVQNTISYEQPDFRTIQRKDDANLASWDIKFVETKDGYNIDSYHAIYGAEAAAKEAAAKEAAAKEAAAKAIPVEKVWVGQTSERAEIKLFADGIEVDKVILNADNNWKHTFENKPEYNSETKQKINYSVSETTISGYESNITGDAKNGFIVTNTELPDLTIGKEVIGELGDKTKVFNFELTLKQADGKPINGKFNYIGSVDDRYKKESIKPSDGEITFIEGKATITLSHGQEITIKDLPYGVTYKVMEKEANENGYLTTYNGNNEVTTGELAEAAAKEAAAKEAAAKEAAAKADPSVGKNVKELVAYISTSGEKDAGTDDYMYFGIKTKDGKTQEWEMDNPGNDFMTGSKDTYTFKLKDENLKIDDIQNMWIRKRKYTAFSDAYKPENIKIIANGKVVVDKDINEWISGNSTYNIKAEAAAKEAAAKEAAAKEAAAKALVDGEEKFNSGVMRSTTPQKYVKVDVKNAKELKLIVNDAGDGDSSDHASFGDAKLATLSSKPIIKGENLAYSMDEKVDLMKGITATDIEDGNITSKVQIKSSDFVEGKSGIFTVVYSVTDSDGLTSECSRTIAVTDKETQLSDLNWKSATIGSGSVRKDRAVSGNQIRLLNEDNSVETFAKGIGT**HHHHHH**..**

The protein was approximately 77 kDa and expressed in an IPTG-inducible plasmid pET30a. The his-tagged protein had a purity of ~85% and a concentration of 0.5 mg/mL. The SDS-PAGE and Western blot results supplied by GenScript are displayed in Figure 6S5.


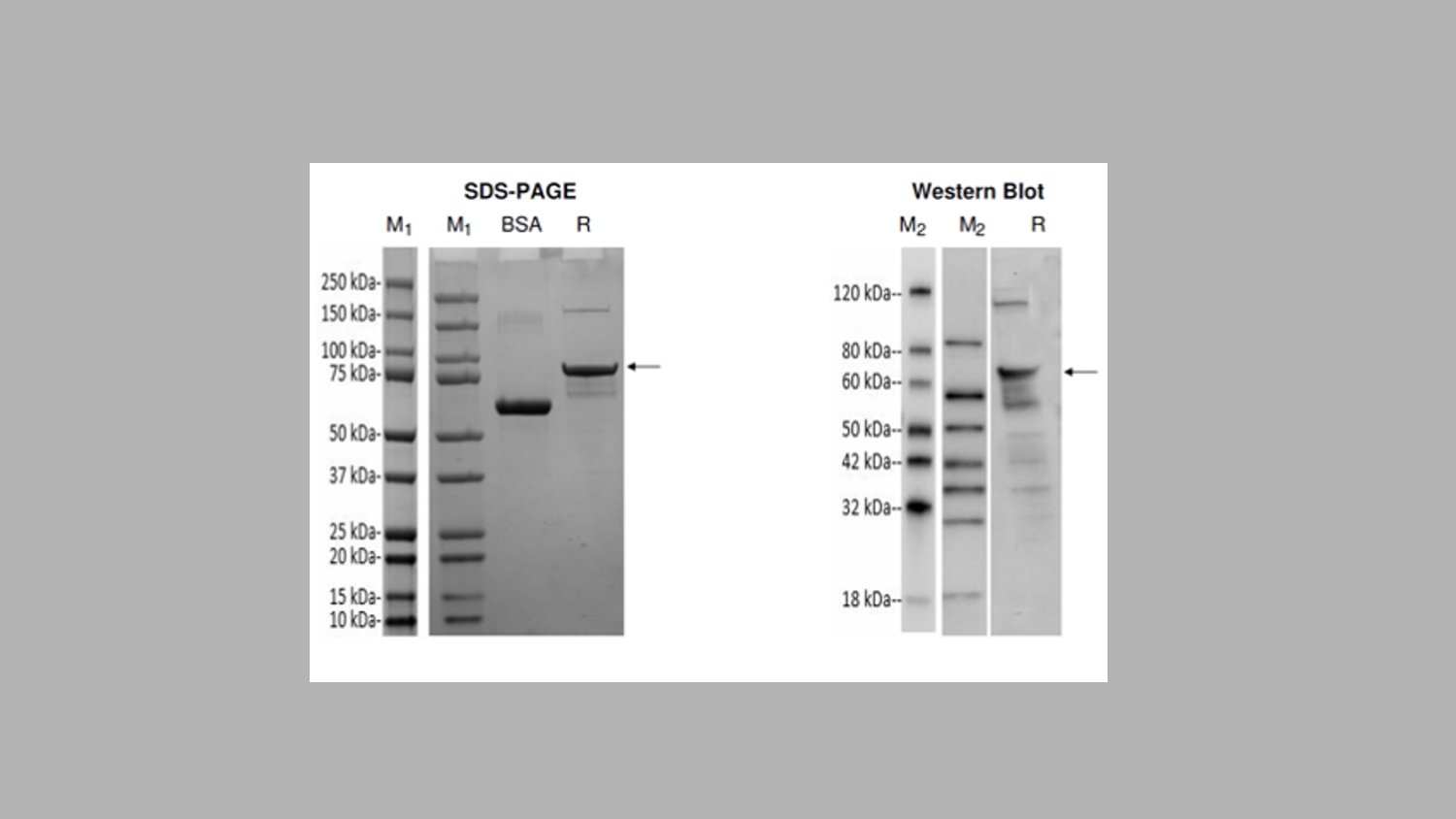


**Figure 1S. Protein gel electrophoresis results of QV-protein.** Lane M1 and M2: Protein marker (GenScript Cat. No. 1610374S, Cat. No. M00521), BSA: Bovine Serum Albumin (2 µg), Lane R: Quadrivalent protein (indicated by arrows, approx. 77 kDa, reducing conditions). Primary antibody: Mouse-anti-His mAb (GenScript, Cat.No. A00186).
